# “Will I be able to be myself? Or will I be forced to lie all the time?”: How Trans and Non-Binary Students Balance Professionalism, Authenticity, and Safety in Canadian Medical Programs

**DOI:** 10.5334/pme.1199

**Published:** 2024-02-20

**Authors:** Kat Butler, Meredith Vanstone, Adryen Yak, Albina Veltman

**Affiliations:** 1 Department of Anesthesiology and Pain Medicine, University of Toronto, Toronto, CA; 2 Department of Family Medicine, McMaster University, Hamilton, CA; 3 Family physician in Toronto, CA; 4 Department of Psychiatry & Behavioural Neurosciences, McMaster University, Hamilton, CA

## Abstract

**Introduction:**

Promoting the inclusion of trans and non-binary (TNB) medical trainees is a key step in building an inclusive health workforce well-positioned to provide high-quality healthcare to all patients. Existing data on the experiences of TNB physicians and trainees describe widespread challenges related to prejudice and discrimination, with most trainees concealing their gender identity for fear of discrimination. We aimed to understand how TNB medical students have experienced professionalism and professional identity formation.

**Methods:**

This was a secondary analysis of data gathered in a constructivist grounded theory study. The authors conducted semi-structured qualitative interviews in 2017 with seven current or recently graduated TNB Canadian medical students.

**Results and Discussion:**

From medical school application to graduation, TNB medical students reported feeling tensions between meeting expectations of professionalism, being their authentic selves, and seeking to avoid conscious and implicit biases. These tensions played out around issues of disclosure, foregrounding identity through impression management, and responding to identity exemplars. The tension between TNB trainees’ desire to bring their whole selves to the practice of medicine and feeling pressured to de-emphasize their gender is ironic when considering the increased call for medical trainees from equity-seeking communities. The most commonly used behavioural frameworks of professionalism were inherited from prior generations and restrict students whose experiences and community-based knowledge are most needed. Demands of professionalism that are incompatible with authentic professional identity development place an inordinate burden on trainees whose identities have been excluded from normative concepts of the professional, including TNB trainees.

## Introduction

Trans people are individuals whose gender is “different, discontinuous, or more complex” [1] than the gender that is culturally aligned with the sex assigned to them at birth. The language used by gender diverse people to self-identify continues to evolve [2]; however, in this paper, trans and non-binary (TNB) will be used as an umbrella term for the many gender identities included in the above definition. The inequities facing TNB people seeking healthcare have been well-documented [3,4], as has the role of clinician bias and under-preparation in the care of trans patients [5,6,7,8,9]. As with other underserved groups in medicine, one approach to redress these inequities is to increase the number of TNB clinicians [10,11,12,13]. In the last decade, literature about TNB medical trainees has been gradually increasing [13,14,15,16,17,18,19]. Data from the AAMC shows that TNB matriculants in US medical schools increased from 0.5% of the student population in 2015 to 1.4% in 2023 [20,21]. In Canada, where this study was conducted, demographic surveys estimate that approximately 0.2–0.8% of medical students identify as TNB [22,23]. While representation is increasing, the existing data on the experiences of gender minority physicians and physicians-in-training paints an unappealing picture for any hopeful student. Sixty percent of TNB medical students report concealing their gender identity for fear of discrimination and lack of support. More than two-thirds of TNB medical students and physicians in medical school, residency, or practice have witnessed derogatory comments about trans people; a third of these TNB medical students and physicians have witnessed the discriminatory treatment of a trans patient [18].

One site of potential distress for TNB medical students is the interface between gender identity and professionalism. Professionalism in medical education has been constructed through multiple iterations—both uncritically and critically—and continues to be used as a tool to control trainee behaviours, particularly when a behavioural model of professionalism is enacted [24]. Several studies describe how professionalism constructs can be wielded to perpetuate racism, sexism, heterosexism and other forms of discrimination though the differential enforcement of subjective professionalism standards across learners [25,26,27,28]. Given a history of deeply gendered discourses on professionalism, the experiences of TNB students offer unique insight into the way that these policies may serve to enforce conformity with cisgender (non-trans) norms. Though there has not yet been any published data on the experiences of TNB medical trainees’ experiences with professionalism, data from other groups historically excluded from medicine would suggest that professionalism might similarly be weaponized against TNB trainees.

Professional identity formation (PIF) is a concept which has been proposed in contrast to traditional approaches to teaching and learning professionalism [29,30]. This concept has been operationalized in many different ways, characterized by a focus on the evolution of a trainee’s identity through the process of acculturation into a medical professional identity, with a strong emphasis on the developmental trajectory of a learner [31,32]. PIF theories also usually acknowledge identity development as a continuous process, starting prior to entering medical training [31,33], rather than viewing new trainees as blank slates [34]. In this analysis, we aimed to understand how TNB medical trainees experience the development of their professional identities, leaning on a social-contextual approach to professional identity formation to understand the ways in which identities are formed through social interaction within and outside of a medical milieu [35]. This paper describes the ways TNB medical students worked to develop authentic professional identities while balancing behavioural codes of professionalism with safety from discrimination during their training, yielding insights into the constraining effects of policies and expectations related to professionalism.

## Methods

This is a secondary analysis of data gathered in a constructivist grounded theory study of the experiences of TNB medical students [36]. The methods of this study have been previously described [15]. The initial study was focused on understanding TNB medical students’ experiences of undergraduate medical training in Canada; this analysis focuses on the question of how TNB medical students experienced professionalism and PIF. This question arose through the analysis of the initial dataset, as participants came back to this topic repeatedly when responding to multiple interview questions.

Between April 2017 and April 2018, we conducted semi-structured interviews with current or recently graduated Canadian medical students who identified as TNB recruited via closed online groups for Canadian SGM medical students and through SGM health research networks. Participants discussed their experiences applying to medical training, academic and clinical experiences, interactions with administration, curricular inclusion of TNB health topics, access to mentorship from TNB faculty, experiences accessing healthcare, and career planning. These interviews lasted 60–90 minutes and were audio-recorded and transcribed verbatim. We used a staged process of analysis common in grounded theory, which began with open coding and moved to focused coding. At the time of data collection, KB, AY, and AV were medical trainees or staff physicians belonging to TNB and/or queer communities; as such, we approached the interviewers as community insiders. We implemented reflective memo-writing, peer debriefing, and member-checking, and requested that participants elaborate any statements that implied a shared understanding during interviews [36,37]. While a grounded theory approach does not encourage the use of a prior theory to inform analysis, we recognize that the identities, experiences, and knowledge we brought as scholars, clinicians, and people informed this work. Specifically, our familiarity with various theories of professional identity formation, gender, and social power informed our understanding of the data, drawing attention to the social and cultural ways in which our participants navigated their medical education and formed their professional identities while also negotiating a traditional, normative, and hierarchical space [38].

This study was reviewed and approved by both the Hamilton Integrated Research Ethics Board and the McMaster University Undergraduate Medical Education Protocol Review Committee. Notably, given our small sample size and concerns for identifiability, we do not attribute quotations to specific participants in this manuscript, or provide a table with disaggregated participant characteristics.

## Results

Seven TNB medical students participated in this study, and their demographic features are included in Table 1. Analysis of the data indicated that all participants expressed the tension between meeting expectations of ‘professionalism,’ being authentic, and seeking to avoid both conscious and implicit bias from others (see Table 2 for themes and subthemes). This tension was present from the application and admissions process for undergraduate medical programs through to clinical experiences and application to residency, and participants described a number of approaches to finding this balance. In the sections below, we describe how the tensions of professionalism, authenticity, and safety were navigated at each stage of medical learning.

**Table 1:** Participant demographics (reprinted with permission from Butler) [15].


DEMOGRAPHIC CHARACTERISTIC	RESPONSES

Gender identity^1^	

Genderqueer	4

Non-binary	2

Genderfluid	1

Agender	1

Trans man	1

Woman	1

Region	

Québec	3

Western Canada	2

Ontario	1

Eastern Canada	1

Age	

26–30	5

20–25	2

Accessed legal name or gender marker change?	

Yes	4

No	3

Sought trans-specific health care? (e.g., hormones or hormone-blockers, surgical intervention, voice therapy)	

Yes	3

No	3

Not yet, but considering it	1


^1^ Several participants indicated multiple gender identity descriptors.

**Table 2:** Analytic schema, listed by theme.


**Disclosure in applications and admissions**	Uncertainty regarding how admissions committees will view trans and non-binary identity/activities Disclosing vs. not disclosing gender identity in applications & interviews Reflections on applications & interviews Role of conscious & implicit bias in admissions and applications

**Backgrounding/foregrounding identity through impression management**	Clothing and physical presentation How others perceive/ interpret gender Disclosure – to classmates, preceptors, patients Coping with comments Backgrounding/foregrounding identity Ideal world vs. real world “Weird” Non-binary experiences “What else could I be spending time on, if I wasn’t thinking of this?”

**Clinical experiences and evaluations**	Evaluations Uncertainty Preceptors Managing safety & vulnerability

**Responding to positive and negative exemplars of professional identity**	Negative experiences Exemplars of advocacy and care Mistreatment in clinical settings


### Disclosure in applications and admissions

Admissions processes presented applicants with the dilemma of whether and/or how to disclose or disguise their gender identity. While most participants did not disclose their gender minority status on their applications to undergraduate or graduate programs, specifically out of fear of discrimination, two participants did disclose their gender identities in undergraduate admissions. One individual disclosed within applications, and the other during interviews:

I was actually not sure if I should be open about my real reasons for wanting to do medicine–like how it was tied to my gender identity, my experience transitioning–because I guess my perception of medicine and healthcare was that it was pretty conservative…I was pretty honest about it with all my applications.

In some instances, participants discussed how disclosure of TNB identities and the inclusion of SGM-related activities on their applications might provoke bias from file reviewers. Three participants talked about their approaches to interviews as more focused on authenticity within the constraints of presenting a professional image.

Then for the interviews [pause] you know, I think it’s always a question of what being professional is and looks like, and how you come off. I think for me, I guess I see myself as pretty pragmatic, so my goal in interviews was not to disguise myself in terms of who I was, to suddenly look more gender conforming–but, you know, wearing nice pants, nice suit, nice clothes—looking professional while still not changing too much who I was.

One participant discussed the specific vulnerability of being transfeminine in these high-stakes contexts:

I sort of chose the sort of male professional role, since that’s sort of non-threatening. I think that even some people who think of themselves as allies will feel sort of uncomfortable around people who are presenting as transfeminine, so I was sort of hedging my bets by making the decision to wear a suit and tie.

Disclosure of identity was also relevant in the selection of professional contacts (“referees” and “verifiers”) who provide reference letters or other official attestations about an applicant’s participation in an activity. Participants who had undergone a name change, gender marker change, and/or transition since their participation in activities listed on their applications described a great deal of stress in identifying referees and verifiers. Participants used a variety of strategies in this process, ranging from simply hoping that verifiers from high school activities would not be contacted, to specifically asking referees who were aware of their TNB identity not to make any reference to it.

### Foregrounding/backgrounding identity through impression management

Several participants talked about the need to navigate how they presented their gender identity, giving examples of foregrounding or backgrounding certain parts of their identities, depending on the situation and sense of relative safety. Most commonly, this was a movement to background TNB identities (e.g., not correcting people when they used an incorrect pronoun, making careful clothing choices). This process of impression management, or attempting to shape the perception that others have of you, was particularly complicated by expectations of professional attire:

When it comes to professional clothes, there is such a binary of what is considered professional. There’s a masculine professional look, and there’s a feminine professional look—there’s no in-between. There’s nothing that you can wear that makes it so that you’re read as someone in-between or neither. I had such trouble with that—not identifying as either, I always had to pick a side. So, it never seemed true, it never seemed genuine.

In their visual presentations of self, participants discussed the tenuous balance of professionalism, authenticity, and avoiding bias. One participant described how wearing a white coat was key to managing a professional image.

I just tried all the time to be professional so that no one could say anything about me, or give me a poor evaluation or criticize me based on how I looked. I always made a point of wearing my stupid white coat—dressing professionally, and being on time, things like that.

Prioritizing professionalism (i.e., conformity to rules and norms) over authenticity resulted in the commonly described experience of being perceived as cisgender by their classmates, preceptors, and patients. This perception was often seen as a source of protection from overt discrimination but also led to a continued sense of invisibility and exposure to transphobic comments.

So, it’s frustrating because I’m trans, but people don’t read me as trans, so on the one side, I have this privilege of not being discriminated against, but on the flip side, people are much more open about being transphobic around me…I have constant reminders that I don’t fit in.

Some participants did choose to disclose their gender identity in particular situations where it felt safer, such as when a preceptor asked for pronouns or when working with a trans preceptor:

I’ve had a couple of preceptors ask me what my pronouns were in the context of offering to write reference letters. And I am simultaneously thrilled and a bit terrified when they ask this question because a) I’m so happy they’re asking, because it means maybe they’ll ask their patients, and maybe they’ll ask the next learner and b) who knows what a reference letter with gender-neutral pronouns will look like to program directors for [residency applications]? Anyway, I really appreciated it both times and told them my pronouns.

Six participants spoke at length about the process of balancing professionalism, safety, and authenticity regarding impression management—and the taxing nature of navigating these elements.

### Clinical experiences and evaluations

Several participants described the beginning of clerkship as a time when the tension between their own identity and a professional identity became more strained, as they began to negotiate the day-to-day process of interacting professionally in constantly changing clinical settings. This transition to a clinical professional role was accompanied by anxiety and uncertainty for several participants, especially regarding authenticity and safety. One participant described their anxiety as they approached clerkship:

Will I feel obliged to come out every month? How will I be perceived? If there’s a preceptor who doesn’t like me or isn’t okay with [me being trans]. Will I be able to be myself? Or will I be forced to lie all the time, every month?

This concern regarding disclosure of trans identities was not explicitly addressed by most participants, as most avoided disclosure of gender identity to preceptors. Reasons for this avoidance included feeling unsafe disclosing their gender in clinical settings, not wanting to be perceived as a ‘difficult’ student, and fearing bias, particularly regarding evaluations.

Participants emphasized the role that preceptors played in mediating their experiences in clinical settings, particularly in relation to the perceived subjectivity of evaluation processes and biases surrounding what constitutes professional or unprofessional behaviour. Several trainees expressed trepidation about the ‘incontestable’ nature of evaluations and fear that a preceptor’s bias against TNB trainees could lead to negative evaluations of their perceived professionalism.

Of course, there is the sort of vulnerability of being a medical learner, where you’re being graded in a very subjective way…if they were to give you a bad evaluation based on something other than your performance, then there’s no real way to challenge that. So as a medical student, because the evaluations are so important, I think that there is sort of a threat, and then it makes it very difficult to stand up to preceptors or even other classmates or residents.

### Responding to positive and negative exemplars of professional identity

Alongside these experiences of feeling constrained by the potential biases of preceptors, several participants related positive experiences witnessing clinicians caring competently for trans patients. These positive interactions served as exemplars of clinicians who integrated competent care for trans patients as part of their professional identity. They also served to demarcate clinical spaces in which participants could prioritize authenticity in their own personal and professional identities. Participants consistently expressed surprise and joy at witnessing these encounters, particularly in non-TNB-focused settings, thus highlighting the exceptionalism of trans-positive care.

I was very surprised by how they approached the patient. The nurse actually advocated for the patient to return to estrogen therapy, and acknowledged to the patient and to the doctor how important it was for the patient…The doctor was respectful towards the patient about being trans and very respectful of the patient’s desire to continue the hormone replacement therapy.

In line with previously published medical student perspectives on professionalism, there is a power-inflected double standard of acceptable behaviour for students and attending physicians. Several participants reported experiencing and witnessing interactions with staff physicians that bordered on mistreatment; one participant witnessed their attending physician speak disrespectfully about a trans patient, while two others described very negative experiences with preceptors treating them disrespectfully. Constrained by a fear of reprisal, and choosing to prioritize safety over authenticity, neither of these participants formally addressed their experiences. One trainee described their protective strategy for dealing with the possibility that their experience might have been influenced by racism, homophobia, and transphobia; nevertheless, they resisted attributing their mistreatment to their core personal identities:

I would prefer to think that this thing happened because this person…is very inappropriate, rather than I was targeted because of my identity or how I present for two reasons: 1. many of my classmates who are straight and cisgender have those experiences where they cry in a bathroom; then 2. it’s just a little more empowering for me personally to be like, ‘I was treated this way because this person is shitty rather than I was treated this way because of who I am.’ Because it’s something that I don’t want to carry with me, like something that is problematized.

## Discussion

TNB medical students described the complex process of navigating conflicting concepts of authenticity, safety, and behavioural codes professionalism as they progressed through training, which stymied the development of authentic professional identities as physicians. Most consistently, participants implicitly understood professionalism in a behavioral framework, wherein the professional competencies of a trainee are assessed externally on criteria such as appearance, communication, and punctuality. This is consistent with the way professionalism is operationalized at many medical schools in Canada. ‘Professionalism flags’ continue to be put forward for a range of offences—from tardiness to making racist remarks to taking part in protests against racism [25,39]—and, as echoed in this study, medical students continue to experience this enforcement of behavioural forms of professionalism as a threat [39].

Participants marked this behavioural enactment of professionalism as a particularly constraining force to identity formation, particularly in high-stakes transitional contexts, including undergraduate and graduate medical admissions processes and the beginning of clerkship or of new clinical rotations. They acutely felt the limits of these expectations when making decisions about personal appearance, engagement with their communities, and decisions about whether to speak up when colleagues, supervisors, or patients expressed biased behaviour towards TNB patients.

Social-contextual theories of professional identity formation (PIF) help us understand some alternatives to the enactment of behavioural codes of professionalism [29]. PIF has been conceptualized in many ways, characterized by a focus on identity formation as a dynamic process that occurs over time, informed by both individual and social aspects [30,35]. Identity theories informing these frameworks draw attention to various aspects of identity formation. Particularly relevant here is self-categorisation theory, which helps us recognize how particular aspects of an individual’s identity become relevant at different times, as they move through different social groups and learning contexts [35,40]. In this data, TNB participants consistently felt their gender identity to be relevant in their navigation of particular professional situations, and identified challenges integrating multiple elements of their identities, an essential component of PIF. Medical students with identities that are incongruent with the profession’s historical identity (i.e., white, male, high socioeconomic status) have been described as experiencing identity dissonance through the process of PIF, as fundamental parts of their personal identities are brought into conflict with the explicitly espoused values of medical professionalism [41,42]. In this study, there was frequently a drastic dissonance between the personal identities of participants and the expectations of professionalism which they saw modeled and described in their academic and clinical settings. The tension wrought by this dissonance led participants to engage in behaviours that they described as simultaneously self-protective and inauthentic.

Yoshino describes ‘Covering’, a concept adapted from Goffman, as the adoption of normative behaviours by people with non-normative identities to fit in with normative expectations, usually as a way of avoiding discrimination [43,44]. Covering is understood as a barrier to authenticity and, therefore, to any real acceptance of the broad—and normal—variation of humanity. Depending on the degree of risk in any given situation, most trainees in this study engaged in practices that backgrounded parts of their identity that they perceived as unwelcome or dissonant; dressing conservatively during interviews, using names they no longer used in their non-professional lives, and asking referees to avoid mentioning their gender identity. Notably, covering often relies on the heteronormative and cisnormative assumptions of others, not on overt deception. For students in this study, covering sometimes meant not actively foregrounding their identity (i.e., not introducing themselves with pronouns, not asking for access to an appropriate changeroom, etc.). Participants described covering behaviours in contexts in which they felt it was not safe to be their authentic selves.

Authenticity as a concept was raised explicitly and implicitly by participants throughout their interviews. Authenticity can be difficult to define [45]. For these participants, authenticity signified being able to be themselves in clinical and academic settings – represented by the ability to discuss trans-related health topics, advocate for trans patients, dress in ways that felt true to themselves, and have their transcripts and letters of reference reflect their pronouns. Their access to authenticity was severely compromised both by systems that lacked space for their existence and by their perceptions of what was safe in their environments. For several participants, even their underlying motivation to enter medical training, related as it was to their experiences of medical transition, felt too dangerous to discuss in high-stakes admissions interviews.

This tension between the desire to bring their whole selves to the practice of medicine and the need to engage in covering behaviours demonstrates the irony of increased calls to admit students from traditionally underrepresented and excluded communities into medical training. The sharp edges of behavioural frameworks of professionalism retained from prior generations of medical education are now pressing into the students whose experiences and community-based knowledge are most needed to expand the idea of who can be a doctor [10].

For these participants, professionalism and authenticity were seldom reconcilable. How then, did TNB learners respond to this tension as they worked to develop professional identities as physicians? One strategy used by participants was that of resistance, a strategy previously noted in the literature to be in widespread use by medical trainees to respond to ‘professionalism lapses’ (defined as “unprofessional, unethical, or immoral ways of acting”) by their seniors [27]. As described, some learners refused to attribute experiences of mistreatment in clinical settings to their own core identities, instead noting that these experiences of mistreatment were unfortunately common amongst their non-trans peers [46], and problematizing the character of the mistreating clinician rather than their own identity [47]. Resistance to problematizing TNB identities in a professional identity context parallels ongoing community resistance to the medical pathologization of trans people (as exemplified by the ongoing discourse about the harms versus the utility of having a diagnosis for gender dysphoria or previously, gender identity disorder, which simultaneously pathologizes gender diversity while enabling access to medical transition-related interventions [48]). This resistance to the problematization of TNB identities in the realm of medical professional identity could be accompanied by an integration of positive and non-normative examples of professional identities and interactions exemplified by both TNB physician-teachers and those clinicians who took the care of TNB patients seriously. For medical learners to do this effectively, however, acknowledging the history of the construct of professionalism and its enforcement is required, in order to be able to trace the roots of professionalism to their origins in racism, sexism, and colonialism [25,49].

Our findings are specific to the socio-historic context in which they were gathered. The study was limited by its small size, which did not allow for purposive sampling; therefore, there may be both sampling and volunteer bias. Given evidence that 0.2 to 0.8% of Canadian medical students are trans or non-binary [22,23], and there were approximately 11,000 active Canadian medical students at the time of recruitment, we estimate that this study includes 10–30% of the eligible individuals [50]. Similarly, although the initial dataset was not collected to address the topic of professionalism and PIF, in discussing their experiences as TNB medical trainees, participants frequently spoke about professionalism, thus initiating this secondary analysis.

We are also limited in our ability to generalize our results as study participants were mostly homogenous (i.e., identifying as trans men or NB, but not trans women; racialized, but not Indigenous or Black); this is of particular note, given that harmful constructs of professionalism have frequently been applied to racialized professionals and trainees [26]. These limitations are undoubtedly exacerbated by the implicit and explicit exclusion of Black and Indigenous people from medical training and practice in Canadian medical institutions [51,52], and the high degree of discrimination trans women face in professional settings as compared to trans men and non-binary people [53].

Future questions for consideration include: which aspects of professionalism are part of the day-to-day impression management everyone performs in workplace settings, and which are rooted in racist, sexist, ableist, and cisnormative expectations of how a doctor ‘looks’ and ‘acts’? How does normative professionalism undergird the power differential present in exchanges between patients, physicians, and other clinicians—and in what way do these protect or harm interpersonal relationships in the team? What role does developing an authentic professional identity play in career longevity and protection against burnout? These questions may be used to prompt medical educators to re-evaluate how professionalism should be used and enforced in medical training.

## Conclusions

The continued use of professionalism as a threat and enforcement construct limits the ability of medical education culture to meaningfully change. The burden of covering in the face of demands of professionalism that are incompatible with authentic professional identity development places an inordinate burden on trainees whose identities have been explicitly or implicitly excluded from normative concepts of the professional, including TNB trainees. If the widening disparities in health outcomes for TNB people are to be truly addressed, we must work to refigure medicine’s concept of professional identity so that all people may in fact ‘look like a doctor’ and TNB people may benefit from the powerful experience of seeing themselves reflected in the physicians caring for them. As we continue to strive to better serve patients by increasing the numbers of clinicians from communities historically excluded from medical training, we need to consider that our current practices of enforcing professionalism are likely doing more harm than good.

Building an inclusive medical profession that can provide care for TNB people competently requires respect, representation, and the recognition of their inherent dignity. The behavioural frameworks of professionalism currently enforced in training programs are shaped by historical constructs of gender (and race, class, ability, etc.); these traditional structures often preclude TNB people from a sense of belonging or safety. As medical education theory moves towards curricula focused on fostering inclusive professional identity development, there must also be a shift away from rigid behavioural expectations. We must understand that, as the community of physicians becomes more inclusive in its membership, so too must their professional identities.
